# Barriers to accessing health care services: a qualitative study of migrant construction workers in a southwestern Indian city

**DOI:** 10.1186/s12913-020-05482-1

**Published:** 2020-07-06

**Authors:** Maija Santalahti, Kumar Sumit, Mikko Perkiö

**Affiliations:** 1 Master of Social Sciences, Social Policy, Faculty of Social Sciences, Tampere University, Tampere, Finland; 2 Prasanna School of Public Health, Manipal Academy of Higher Education, Manipal, India; 3 Senior Research Fellow, Global Health and Development, Faculty of Social Sciences, Tampere University, Tampere, Finland

**Keywords:** Internal migration, Health care access, Occupational health, Construction work, Distrust

## Abstract

**Background:**

This study examined access to health care in an occupational context in an urban city of India. Many people migrate from rural areas to cities, often across Indian states, for employment prospects. The purpose of the study is to explore the barriers to accessing health care among a vulnerable group – internal migrants working in the construction sector in Manipal, Karnataka. Understanding the lay workers’ accounts of access to health services can help to comprehend the diversity of factors that hinder access to health care.

**Methods:**

Individual semi-structured interviews involving 15 migrant construction workers were conducted. The study applied theory-guided content analysis to investigate access to health services among the construction workers. The adductive analysis combined deductive and inductive approaches with the aim of verifying the existing barrier theory in a vulnerable context and further developing the health care access barrier theory.

**Results:**

This study’s result is a revised version of the health care access barriers model, including the dimension of trust. Three known health care access barriers – financial, cognitive and structural, as well as the new barrier (distrust in public health care services), were identified among migrant construction workers in a city context in Karnataka, India.

**Conclusions:**

Further qualitative research on vulnerable groups would produce a more comprehensive account of access to health care. The socioeconomic status behind access to health care, as well as distrust in public health services, forms focal challenges for any policymaker hoping to improve health services to match people’s needs.

## Background

India has achieved substantial development in the last couple of decades to reach universal health coverage, which includes expanding the coverage of public health care to those who are unable to use the services [1–3]. A high-level expert group on universal healthcare coverage was founded in 2010 by the Planning Commission of India and tasked with the responsibility of providing a framework for the execution of universal health coverage in India. The National Urban Health Mission was launched in 2013 to target the vulnerable sections of the urban population—which included migrant construction workers and temporary migrants, with the vision of providing universal health care coverage and setting quality standards for service providers, as well as quantitative standards for service provision based on town size [4]. There is a need to assess how vulnerable groups in India perceive their access to health services. This study presents the narrative of the migrant construction workers in a Southwestern Indian city.

As per the latest data available from the year 2011, India has more than 450 million internal migrants, which is more than a third of its entire population [5]. Better employment and education opportunities in urban areas have attracted migrants, particularly men, from rural areas in India. Unemployment is the leading cause of migration, after marriage and moving with one’s household. Geographically, the nature of development and industrialisation in India is sporadic. The provinces of Western and Southern India are industrially more developed than their Northern and Eastern counterparts, and this impacts the direction of the interstate migrant flow. However, the majority of internal migration in India happens within the states, as only about 13% of internal migrants were interstate migrants in 2011 [5]. Women’s migration is connected to caste, cultural norms of the family life and the skills needed for work. Women of low social status are more likely to migrate for work than those who are from affluent social backgrounds [6].

Rural to urban migration is the dominant form of migration for people moving between Indian states [5]. Migration to urban areas is largely due to the ongoing rural distress in many parts of rural India [7]. To this end, in many parts of India, there has been a downward spiral in agriculture due to prolonged drought [8]. In their study among internal migrants in Bangalore, Karnataka, Premchander et al. found that landlessness or small landholding, poor employment opportunities and low wages were the most important reasons for migration [9]. The construction sector is one of the most significant labour sectors in India and the second-largest sector of unorganised labour after the agricultural sector. Between 2009 and 2010, there were 44 million workers in the construction sector [1].

This study was conducted in the university town of Manipal, which is a suburb of Udupi District in Karnataka, India. The majority of the residents in the town are university workers or students. There is a booming construction sector in Manipal, which requires workers, many of whom move from other parts of the country. After the liberalisation of the Indian economy in the mid-90s, Karnataka has experienced rapid economic growth, mainly due to the expansion of the IT sector [10].

India’s current social security provision is directed towards formally employed workers or officers of either the public sector or organised private sector establishments, which leaves large swathes of the working population unprotected [11]. Organising social security schemes is challenging because workers in the informal sector do not have documented long-lasting employer–employee relationships and their work is temporary and seasonal. Gideon [12] documented a linkage between vulnerable employment and access to health services in Chile, which has a different social context to India. This study showed that the increase in informal employment and subcontracting of workers meant many employees were excluded from health insurance programmes. This study maps the situation in a specific sector of Indian workers.

The health care access barriers (HCAB) model introduced by Carrillo et al. [13] approaches health care access from an individual’s point of view. The model identifies three different categories of HCABs – financial, structural and cognitive. All three categories are seen to be closely connected. They can cause late presentation, decreased prevention and decreased care, which can lead to health outcome disparities [13]. Migrant construction workers, a population of multifaceted vulnerability, are at risk of lack of treatment and related weakened health outcomes. This heterogeneous group of workers includes both genders and varies in terms of employment arrangements and type of migration. The HCAB model may be seen as less comprehensive than e.g. Andersen’s [14] holistic model of healthcare access. However, as we wished for an individual focus, the HCAB model provides a focused theory fit to test how the studied group perceived challenges in accessing health care.

Access barriers to health care is an international phenomenon. Poverty has been identified as a critical component of low access across low-income countries, highlighting the significance of the financial barrier [15]. This study also documents how low income hinders access to health care. In Australian rural and remote contexts, several structural barriers to health care access such as service availability have been introduced, and geography is intensified with financial barriers (affordability) [16]. A study among Polish migrants in Norway showed both system- and people-related barriers to migrants’ access to health services. The identified barriers include practices, skills and attitudes of personnel, as well as various communication problems that hinder access to health care. In contrast, friendly and equal treatment, as well as the presence of a Polish social network, worked as facilitators to access to health care [17]. Hunter-Adams and Rother [18] discovered high cognitive barriers when they studied communication between South African health care providers and Congolese, Somali and Zimbabwean migrants. Participants had fears over unwanted procedures or being unable to access care. Communication without a common language was associated with frequent use of migrants’ partners as interpreters that caused the loss of income and nonprofessional medical interpretation. This demonstrates how a cognitive access barrier may increase financial barriers, a connection which will be further analyzed in this study.

The purpose of the current study is twofold. First, it aims to explore if the barriers to accessing health care exist among migrant construction workers in an Indian context. Second, the analysis is open to any finding that may arise from the data. Overall, the study seeks possibility to provide a revised version of the HCAB model for a vulnerable group in India that employs masses of people from both genders.

## Methods

### Data collection

The data of this study were collected using semi-structured in-depth interviews (see the interview guide developed for this study as a supplementary file). Topics of interviews were identified beforehand, and the interviews followed a broad thematic structure. The themes included experiences of health care services in respondent’s current locality and their homes, decision making related to health care seeking, knowledge of services, availability and accessibility of health-related information and challenges relating to health care service access. The main reason for the choice of in-depth interviews as the data collection method was that open-ended questions allowed capturing of lived experiences and made it possible to keep the study open for emerging themes throughout the process. The method enabled the theme of distrust in public health care services to emerge.

Choosing a qualitative approach was justified because the aim of this case study was to produce sensitive knowledge of the complex experiences of the participants. The purpose of this study is to offer an analytical generalisation, not a statistical one [19]. However, data collection was linked to the research questions. First, the plan was to collect sufficient data to validate whether the access barrier model supports the views of the vulnerable group studied. Second, once distrust began to emerge, as a result, the interview data needed to be sufficient in order to conclude about distrust. Jennifer Mason [20] stated that the sample size should allow a study to make meaningful comparisons of results and to develop and test the study’s explanation. The final theoretically motivated sample size for this study was 15 respondents. At that point, it was noticed that both the access barrier model as well as the new finding on the distrust of the public health services holds widely among respondents. Malterud, Siersma and Guassora [21] stated that the information power of the sample depends on different aspects. In this study, both the group of interest and the theoretical approach were specified and supported the aim of the study. Hence the information power of the sample was sufficient for meaningful analysis.

### Study population

The interviews were conducted in Udupi, Karnataka, in Southwestern India in January and February 2019. Participants were recruited by convenience sampling and on a voluntary basis. This may result in an overrepresentation of more social and critical persons. The convenient sample also included elements of purposive sampling in order to include both inter- and intrastate migrants, men and women, and migrants of different ages. To increase reliability, participants were recruited from two major construction sites of Manipal and two living areas of migrant workers. Most participants were between 18 and 30 years of age, but there were also older participants up to the age of 62 years. There were both skilled and semi-skilled labourers. Skilled labourers worked as, for example, electricians and masons and semi-skilled labourers as helpers and security guards, among others. Details of the study population are presented in Table 1. All the interviews were conducted with the help of two local students who interpreted the discussion between English and Hindi or English and Kannada. The average length of the audio-recorded interviews was 31 min. The interviewer wrote field notes during the data collection phase.

**Table 1:** Characteristics of Study Population

	Inter-state	Intra-state	18–30 years old	31–50 years old	51 years old and older	Skilled labourers	Semi-skilled labourers
**Men (10)**	9	1	7	0	3	7	3
**Women (5)**	0	5	3	2	0	0	5
**All (15)**	9	6	10	2	3	7	8

### Data analysis

The data gathered was analysed using theory-guided content analysis [22]. The abductive content analysis applied here combined deductive and inductive elements in order to build a complete understanding of the results [23]. The deductive part applied a prior theory – the HCAB model – as an analytical framework for coding and sorting the data. With the HCAB model, experiences of HCABs were sorted into categories of structural, financial and cognitive barriers. The analysis was not limited to the scope of the model. Besides seeking evidence for the model among construction workers, the analysis was open to the themes emerging from the data. Within this inductive part of the analysis, distrust in public health care services was identified as an additional barrier to health care access. In this way, the HCAB model did not only guide the analysis, but itself was also developed during the process.

## Results

**Fig. 1:**
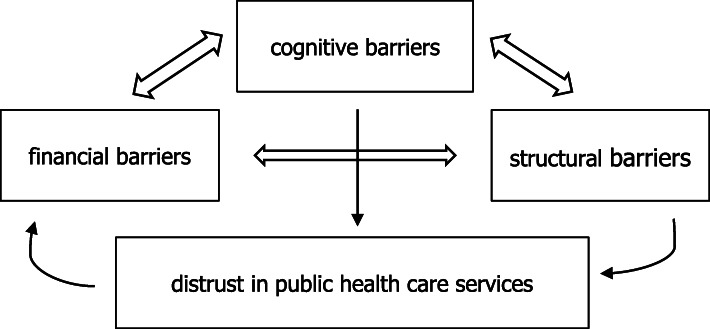
Contemporary Health Care Access Barriers Model (the Authors’ Creation)

All three categories of barriers of the HCAB model – financial, cognitive and structural – were represented in the data in several ways, as was the barrier of distrust in public health care services (see Fig. 1). Despite identifying these different barriers, many workers did not find their overall access to health care unsatisfactory. Moreover, the barriers to access to health care seem to follow social determinants in society; the challenges are related to factors such as income, type of employment and migration.

### Financial barriers

The critical component of financial barriers in the HCAB model is the cost of health care [13]. Although not a strong barrier for all participants of the study, costs were mentioned by some workers, and for them, it appeared to be a significant challenge. As could be anticipated, the price of health care as a barrier was related to the income of a person, as those with lower income saw it as a stronger barrier. Cost as a barrier was also indirectly linked to the level of informality of employment and gender – those in more informal employment settings, as well as all women, earned less and, therefore, the price of health services makes up a more significant portion of their income.

Those in the least formal form of employment were not provided any financial help with regards to health care by the contractor, while the two other groups were compensated for costs related to work injuries. The contractor in the most formal type of employment also paid for their workers’ other medical or health-related costs, paid for sick days off and provided free transportation to health care facilities when needed. Losing income as well as paying out of pocket for medical costs creates financial barriers for those who do not enjoy such benefits.

### Structural barriers

A typical structural barrier in the HCAB model is the distance to the health facility and the working hours of the health facilities [13]. The workers noted that the opening hours of the health facilities did not allow them to visit the facilities after their working hours. The same challenge was present with the so-called health camps ran in the residential areas by staff of public health facilities. In addition, travelling to facilities kept the migrants from working that day, which was problematic. Structural barriers were least problematic for those workers in the least informal type of employment, as they had more support from their employer to access and finance health services. Distance and transportation were barriers to access to public health services in particular, as the workers lived in urban areas where private facilities were also available to them. There was, therefore, no general lack of services in their current locality, although it was noted as an issue in their home villages. Some of the workers from Karnataka were eligible for a below poverty line card issued by the Karnataka as part of the Rashtriya Swasthya Bima Yojana insurance scheme [24]. This allows them to acquire certain services for free or at a lower price. The migrants, however, shared experiences of situations where they were unable to use the card as it was registered at their home village – a challenge caused by migration.

### Cognitive barriers

Lack of information on factors relating to health care or the inability to use such information create cognitive barriers. The participants of this study mostly had little knowledge of the health services available in their area. Those in the least informal type of employment were aware of the Urban Health Center because their employer had informed them about it, but most of the other workers were not aware of this. Information was mostly not acquired from official sources but from informal networks such as neighbours, colleagues and family members. Acquiring and using healthcare-related information was limited by illiteracy and by lack of access to many technologies. These challenges were identified by both men and women, but seemed to be stronger among women. Migration caused a cognitive barrier in the form of language skills. Some workers found it challenging to know about health and health services because they did not speak the local languages.

### Distrust in public health services as a barrier

During data collection, distrust in public health care services was found to be very high among the participants, and during data analysis, it was added to the HCAB model as a fourth category of barriers. Distrust in public health care services stemmed from perceived lack of quality of care and perceived lack of responsibility and interest of the staff. The quality of public health care services was seen as satisfactory, but still as notably worse than the quality of private services. Lack of quality was explained as, for example, issues with the availability of medicine and equipment as well as long waiting times and delays in service. Also, participants of this study felt the staff of public health care facilities show less responsibility for their work and less interest in the wellbeing of the patient than the staff of private facilities. These views on public health services, which were based on both negative experiences and views shared in informal networks, were expressed by respondents as widely known shared facts. While all groups of participants in the study expressed distrust towards public health services, those in the most informal type of employment as well as all women had a slightly more positive view on public services than the other workers.

Distrust in public health care services is caused, in addition to the reasons above, by a number of mechanisms in society. It is based on a broader concept and discourse on the trustworthiness of public services and local government [25, 26]. It cannot, therefore, be solved by only focusing on the issues of quality and staff responsibility, but must be seen as a component of a broader discussion on political and societal trust. Distrust is, however, no inherent feature for only public services. Examples from Somalia suggest that also private health services can be distrusted if they are poorly organised or pressured by social instabilities [27].

### Intertwined categories of barriers

As Fig. 1 illustrates, in this revised HCAB model, all categories of HCAB (financial, structural, cognitive and distrust-related) are connected. First, financial and structural barriers work together to hinder access when health services are only available at times and places that force a person to stay away from work when using them, which directly cuts their income. Second, structural and cognitive barriers interlink, for example, in cases where service providers are unable to provide health-related information in languages that internal migrants can understand. Third, an example of the connection between financial and cognitive barriers is a situation where internal migrants are not aware of public facilities available to them and therefore seek health care in the private sector, resulting in an added financial burden. The three intertwined categories of barriers were expressed widely and equally among participants, and none of them was significantly stronger than the others. Distrust in public health care services, on the other hand, was the most strongly expressed barrier to health care access.

Structural barriers, such as the quality and availability of equipment and medicine and waiting times, and cognitive barriers, such as inadequate counselling by staff members, are factors contributing to distrust in public services as a HCAB. Distrust, on the other hand, creates financial barriers as the clear majority of internal migrant workers choose private services over public ones, thus facing high prices and the inability to access care because of the costs.

## Discussion

This study confirms the HCAB model, as it shows that financial, structural and cognitive barriers all influence the health care access of internal migrants working in the informal sector. However, distrust as an added barrier is needed to understand the broader picture of HCABs. Figure 1 provides an understanding of how broader societal forces, not only components of the health care system in itself, affect health care access of vulnerable populations. Trust is a combination of two components; it includes confidence in the other person’s skills and competences and their positive intentions. In the case of health care services, this means that for a patient to trust the services, they must believe that health care professionals have both the ability and the willingness to work in favour of the patient [28–30]. A patient’s trust is formed based on their experiences, but trust also affects how they perceive further experiences [31].

Previous studies in India have concluded that the socioeconomic status of a person strongly affects their access to health care [32–34]. In the framework of this study, income, literacy and language skills are examples of socioeconomic factors that can either hinder or advance a person’s health care access. This is also a gendered phenomenon, as income level and literacy rates, for example, are lower among women [35, 36]. Migration and informality of employment create additional barriers, thus creating a situation of vulnerability. The existence of health care access barriers among migrant workers is not inevitable. For example, employers could hinder or remove most of the access barriers by providing basic health services near the work site at an affordable price and at accessible times. However, this would not solve the issue of distrust to public health services among migrant construction workers. The ways to tackle this remain a separate major future challenge for policy makers.

The main aim of this study was not to analyze differences between individual characteristic explicitly, rather to verify if the access barriers exist in the sample at large. The qualitative sample of 15 interviews does not indicate exceptionally strong association between certain background characteristic and health care access, apart from the level of informality of employment. The informality of employment can partially be explained by the division of skilled and semi-skilled labour, as skilled labourers were more often engaged in less informal employment arrangements.

The national health survey that compares South Asian countries indicates a surprisingly good access rate to health services for Indian people [3]. Therefore, in-depth studies are needed to validate how access to health services materialise among underserved communities. This study introduces alternative insights into India’s health care access from the migrant and informal workers’ points of view, not often available or considered in research. While the studied case focuses on one vulnerable group, it encourages the study of health care access among other vulnerable groups in India to strengthen understanding of the broader phenomenon of existing social disparities. Despite the fact that this qualitative study found equal presence for all three health care access barriers and strong evidence for the fourth one, there is need for a survey research to specify the prevalence of these barriers among Indian workers in different sectors and states.

The challenges this study faced were connected to the fact that the data collection took place in a context unfamiliar to the researcher conducting the interviews. Challenges in translations and cultural understanding may have resulted in gaps in understanding all of the data. Alternatively, this cultural distance may have also made visible some norms or ideas that would have been left unnoticed by a solely local research team. The messages of different HCABs in the data were sharp and clear.

This study highlights some of the issues vulnerable groups face as regards access to health care. The socioeconomic status of an individual affects their access to health care and health care access is also influenced by social forces, such as low political trust. The role of distrust should be studied further among other vulnerable groups as this study was only able to analyse a group of people, and how distrust influences the health care access of other groups in India or elsewhere is not known. Distrust is a challenge to any policymaker wishing to improve health services to match people’s needs. Overall, the issue of restricted access to health care cannot be tackled separately from other social challenges.

## Conclusion

This study explored HCABs among a vulnerable population in India and was able to identify the role of distrust in public health care services as an additional access barrier among the studied group. The study shows that there are interlinked financial, cognitive and structural barriers in internal migrant construction workers’ access to health care. The finding that distrust is an essential element of health care access is significant because it connects the HCAB model to broader societal forces and processes.

## Data Availability

The datasets generated during this study are not available for the sensitive and personal nature of the information contained. Data may be available upon justified request from the corresponding author with restrictions and following the ethical approval.

## Abbreviation

HCAB: Health care access barrier
